# RLTG: Multi-targets directed greybox fuzzing

**DOI:** 10.1371/journal.pone.0278138

**Published:** 2023-04-12

**Authors:** Yubo He, Yuefei Zhu

**Affiliations:** The State Key Laboratory of Mathematical Engineering and Advanced Computing, Zhengzhou, China; Nottingham Trent University School of Science and Technology, UNITED KINGDOM

## Abstract

Directed greybox fuzzing guides fuzzers to explore specific objective code areas and has achieved good performance in some scenarios such as patch testing. However, if there are multiple objective code to explore, existing directed greybox fuzzers, such as AFLGo and Hawkeye, often neglect some targets because they use harmonic means of distance and prefers to test those targets with shorter reachable path. Besides, existing directed greybox fuzzers cannot calculate the accurate distance due to indirect calls in the program. In addition, existing directed greybox fuzzers fail to address the exploration and exploitation problem and have poor efficiency in seed scheduling. To address these problems, we propose a dynamic seed distance calculation scheme, it increase the seed distance dynamically when the reachable path encounter indirect call. Besides, the seed distance calculation can deal with the bias problem in multi-targets scenarios. With the seed distance calculation method, we propose a new seed scheduling algorithm based on the upper confidence bound algorithm to deal with the exploration and exploitation problem in drected greybox fuzzing. We implemented a prototype RLTG and evaluate it on real-world programs. Evaluation of our prototype shows that our approach outperforms a state-of-the-art directed fuzzer AFLGo. On the multi-targets benchmark Magma, RLTG reproduces bugs with 6.9x speedup and finds 66.7% more bugs than AFLGo.

## Introduction

Fuzzing is the most popular technique that can effectively and rapidly find vulnerabilities in real-world programs. As one of the mainstream fuzzing methods, coverage-guided greybox fuzzing uses coverage information as feedback, and utilizes genetic algorithm to preserve seeds, select seeds and generate testcases, showing great success in thoroughly exploring code of programs under test (PUT). However, coverage-guided greybox fuzzing aims at maximizing the code coverage, rather than exploring specific objective code of interest, which are widely needed in scenarios such as patch testing [1] and 1-day vulnerability detection [2].

To explore objective code, researchers propose directed greybox fuzzing. The existing directed greybox fuzzing gives more attention to seeds that are closer to specific code, and has achieved much progress in areas such as patch testing, crash reproduction [3, 4], automatic exploit generation [5–8], and specific vulnerability mining [9–13]. In detail, these methods in general firstly calculate the distance from the call graph and control-flow graph of the program. Then, they assign the energy to seeds based on the distance, i.e., more test cases will be yielded if the seed has closer distance to the objective code. Finally, directed greybox fuzzing tests PUT with the yielded input testcases.

When there are multiple objective codes to explore, a mainstream strategy used by existing directed fuzzing solutions such as AFLGo [1], Hawkeye [14] is using the harmonic mean of the shortest distances of each target as the overall distance and assigning energy accordingly to seeds. The harmonic mean cannot reflect the accurate distance between the seed and each target. This strategy prefers to test those targets with shorter reachable paths and has some problems in multi-targets scenarios such as 1-day vulnerability detection, since these targets should be treated equally.

Besides, AFLGo and Hawkeye cannot construct the accurate call graph due to the indirect calls and fail to find some reachable paths to the target. AFLGo will miss some indirect call edges and cannot find reachable paths to the targets, while Hawkeye will construct some infeasible paths and mislead the fuzzer to the infeasible paths.

**Fig 1:**
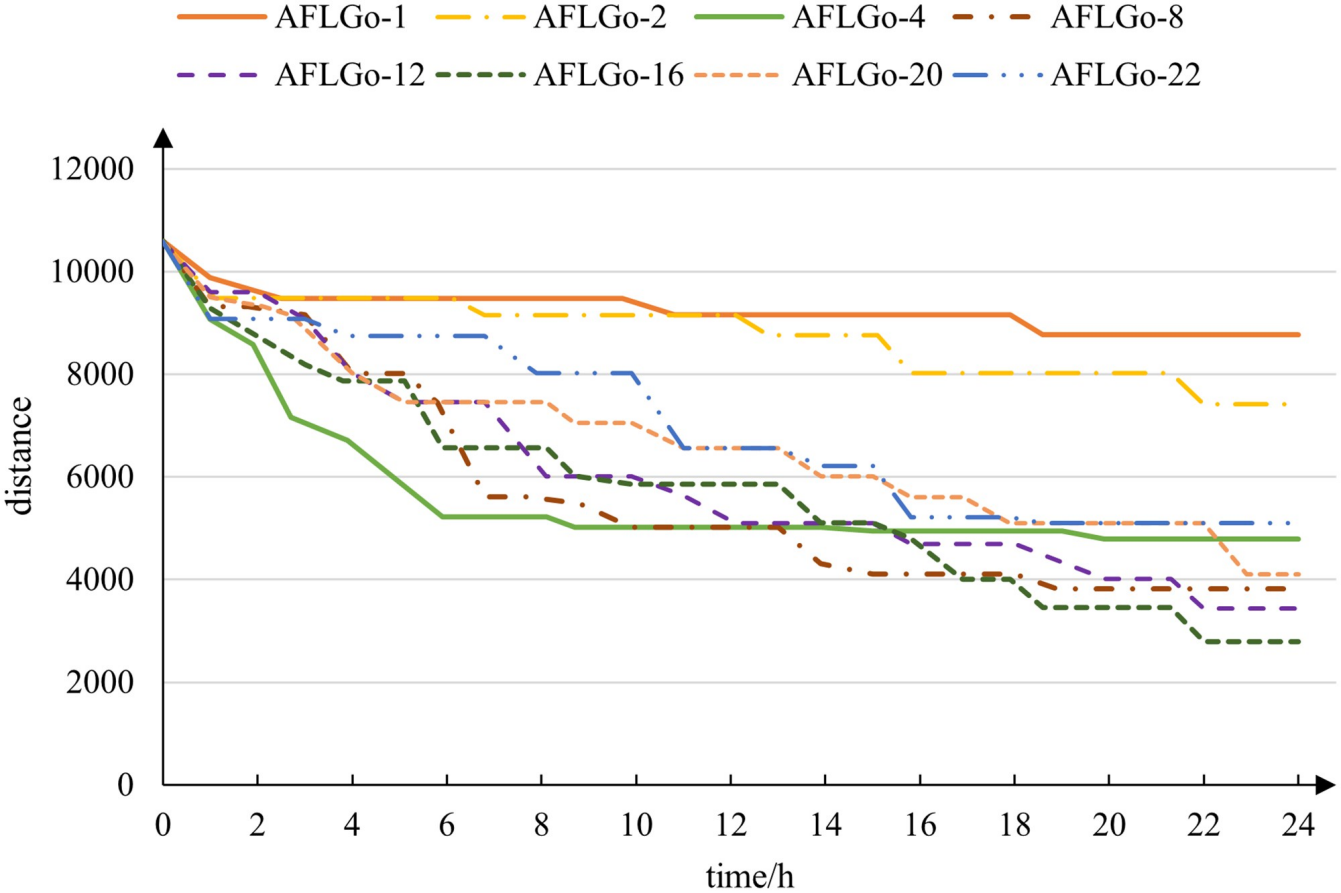
Minimum distance found by AFLGo against different exploration time.

In addition, these methods use multi-priority queues and an option value called exploration time to determine which seed to fuzz and how many test cases should be yielded given a seed. However, the latter scheme has poor scalability, since different programs even different rounds of fuzzing need a different exploration time. As shown in Fig 1 [15], with different exploration time, the minimum distance in the seed pool is also different. The optimal exploration threshold was set to 16 hours since its minimal distance is the lowest, but the default exploration threshold in AFLGo is set to 10 minutes. Besides, using multi-priority queues for seed selection may ignore those seeds with lower access count.

To solve the above problems in multi-targets directed greybox fuzzing, we proposed a dynamic distance calculation method and a new seed scheduling algorithm. First, we found that the existing seed distance calculation methods have different preferences for different targets, and indirect calls in binaries affect the accuracy of the seed distance calculation. Therefore, we proposed a dynamic seed distance calculation scheme to calculate the seed distance. Second, we proposed a seed scheduling algorithm based on the Upper Confidence Bound (UCB) [16] algorithm. This seed scheduling algorithm not only prefers selecting seeds closer to the target but also gives randomly chosen seeds a chance of being selected. Moreover, we argued that the importance of a seed was related to its distance to the targets and its edge code coverage. The former reflects the objectives of directed fuzzing and the latter reflects the possibility of different paths for the seed to reach the target. We design a new seed reward based on this idea and use the UCB algorithm to select the proper seed.

We implemented a prototype of RLTG and evaluated its performance on the Magma [17] dataset and several real-world programs. The experimental results showed that On the Magma benchmark, RLTG can reproduce bugs with 6.9x speedup and find about 66.7% more bugs than AFLGo. On real-world programs, with the help of the seed scheduling algorithm, RLTG can reproduce bugs with 1.65x speedup and find about 60% more bugs.

This paper has the following contribution.

- We propose a dynamic seed distance calculation scheme that can address the indirect call and multi-targets directed fuzzing problems.
- We propose a new seed scheduling algorithm based on the UCB algorithm. This algorithm can address the exploration and exploitation problem faced by existing seed scheduling strategy.
- We design a prototype of the multi-targets directed fuzzing solution RLTG based on AFLGo and evaluated it on Magma and real-world programs. The results showed that RLTG not only can reproduce crashes faster but also can find more bugs.

## Background

### Directed greybox fuzzing

Directed greybox fuzzing is a fuzzing technology that explores specific objective code of interest. Different from coverage-guided greybox fuzzing, directed greybox fuzzer prefers seeds that are closer to specific code and assigns more energy to them for fuzzing. Directed greybox fuzzing selects and prioritizes the seeds using three metrics: distance, sequence coverage, and probability. Of these metrics, distance is a main concern for seed selection. However, distance-based directed greybox fuzzing has difficulty with distance calculation and seed scheduling.

#### Distance calculation

In existing distance-based directed greybox fuzzing, the fuzzer judges the priority of seeds by seed distance. The seed distance is a term used in AFLGo which is calculated based on the distance between functions in the call graph and the distance between basic blocks in the inter-procedural control flow graph. However, when there are multiple targets to reach, existing directed greybox fuzzers prefer the target with shorter reachable path. Besides, existing methods encounter the indirect call problem. To solve these problems, this study proposes a dynamic seed distance calculation scheme.

**Fig 2:**
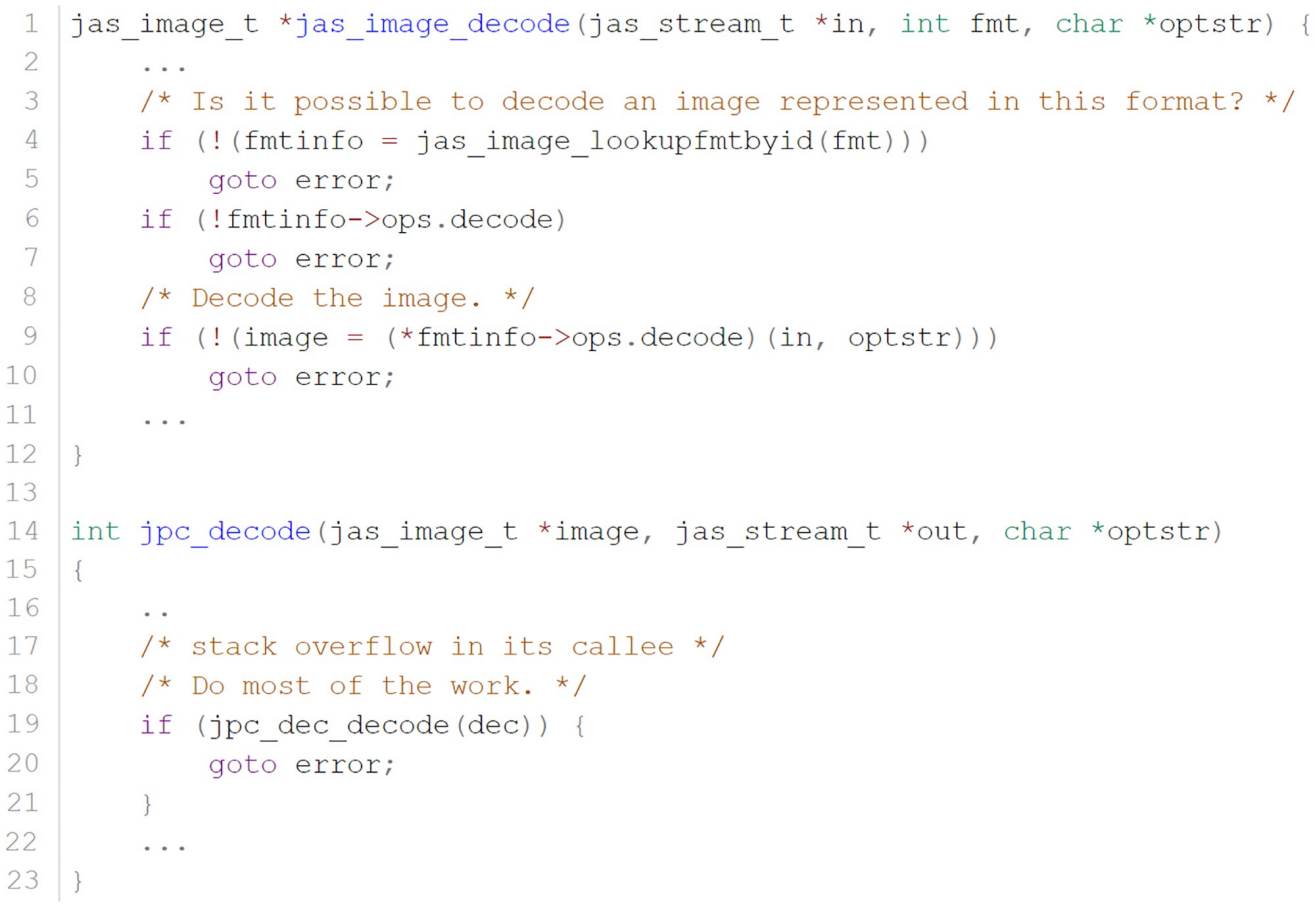
AFLGo may prefer the wrong seed in the program. A is the function entry point, and O and K are the target basic blocks, respectively. A.CFG of program B.execution trace of seed 1 C.execution trace of seed 2 D.execution trace of seed 3.

**Multi-targets distance calculation.** When there are multiple targets to explore, the state-of-the-art directed greybox fuzzer AFLGo donates the current basic block’s distance as the harmonic average of the distances from the current basic block to all the target basic blocks. During dynamic execution, AFLGo collects the executed trace of the seed and calculates the average distance of all the basic blocks in this trace as the seed distance. As shown in Fig 2, in AFLGo, the seed distance of seed 1 is 3.61, the seed distance of seed 2 is 3.63, and the seed distance of seed 3 is 3.09. Thus, AFLGo prefers seed 1 to seed 2, and prefers seed 3 the most because its seed distance is the smallest.

However, since seed 2 reaches the target basic block O, the priority of seed 2 should have been higher than that of seed 1. In addition, seeds 2 and 3 reach the target basic block, and their priority should have been the same. In conclusion, the existing seed distance calculation scheme prefers targets that are closer to the entry function when there are multiple targets to reach, which is the target bias problem in current studies. Therefore, a new distance calculation scheme to accurately calculate the seed distance is needed.

**Indirect call.** An indirect call refers to a function call where the address of the callee is loaded from memory [18]. In distance-based directed greybox fuzzing, distance is calculated on the call graph and the inter-procedural control flow graph. Previous works such as AFLGo and Hawkeye used static analysis to construct the call graph. However, these studies cannot accurately identify the callee of the indirect call, thus these studies cannot build an accurate call graph, which affects the accuracy of the seed distance. Instead, other methods [19] used dynamic analysis to restore the call graph, but these methods depend on the quality of the generated test cases. In addition, the inter-procedural control flow graph will miss some important edges if the initial seed reaching the indirect call site does not call the target callee.

**Fig 3:**
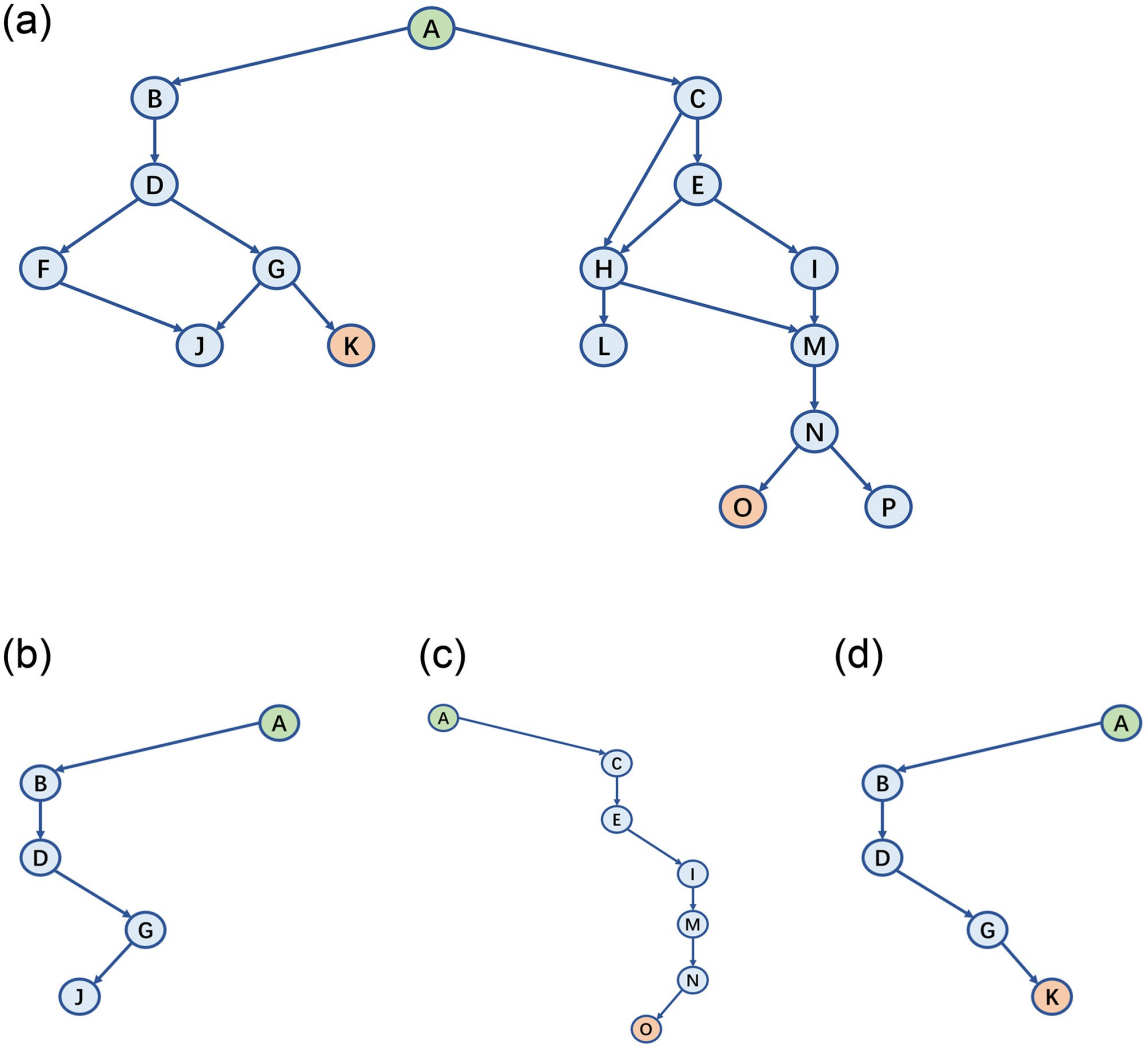
CVE-2015-9560.

As shown in Fig 3, in CVE-2015-9560, there is an indirect call on line 9 that selects the corresponding decoding function with the metadata of the input picture. When the program receives a malicious jpc file, the program calls jpc_encode() to decode the image, thereby triggering a stack overflow in the callee of jpc_dec_decode(). However, previous studies encounter overfit or underfit problems when handling the indirect call in line 9, thus these studies obtain the wrong distance from basic blocks before line 9 to the vulnerability site. Therefore, for the indirect call problem, this study uses points-to analysis to construct the call graph of the program, and prunes the infeasible path during dynamic execution stage.

#### Seed scheduling

The seed scheduling is to select which seed to fuzz in fuzzing process. However, existing directed greybox fuzzers cannot solve the exploration and exploitation problem in seed scheduling.

**Exploration and exploitation problem.** In directed greybox fuzzing, “exploitation” in seed scheduling is to select seeds with lower seed distance, and “exploration” in seed scheduling is to select seed with higher code coverage. The exploration stage is also important in directed greybox fuzzing because the seed with higher code coverage may introduce new program state and help fuzzer find well-performing seeds. The fuzzers need to make a balance between “exploitation” and “exploration” every time fuzzers select seed in seed pool, which is typical of an exploration and exploitation problem.

Traditional distance-based directed fuzzers, such as AFLGo and Hawkeye using simulated annealing algorithm to guide fuzzers to specific code, they provide users with an exploration time option to decide how long the fuzzers enter “exploration” stage after sufficient time of “exploitation” stage. However, these methods fall into the partial optimal solution at initial stage. Besides, different exploration times have a significant impact on the fuzzers, and manually setting the exploration time requires considerable human effort. In this study, we use the multi-armed bandit model to solve this exploration and exploitation problem.

### Multi-armed bandit model

The multi-armed bandit model is a model that describes an exploration and exploitation problem. In detail, the multi-armed bandit mode must solve the following problems: at time *t*, facing a machine with *k* unknown bandits, the object function is *argmax*_*a*_(*R*_*t*_(*a*)), where *a* is the selection of the *i*(*i* ∈ *k*) bandit, and R_*t*(*a*)_is the estimated reward of performing action *a* at time *t*.

Currently, there are many algorithms to solve this problem, such as the ε-greedy algorithm, softmax algorithm, and UCB algorithm [20]. Among these algorithms, the UCB algorithm has best convergence efficiency. At time *t*, the UCB algorithm chooses the bandit with the largest estimated reward, defined as $R_{t}\left(a\right)=Q_{t}\left(a\right)+C\times \sqrt{\left(log\left(t\right)/t_{a}\right)}$, where *Q*_*t*_(*a*) is the estimated average reward of choosing bandit a at time *t*, and *t*_*a*_ is the number of gamblers choosing bandit *a* at time *t*.

Although seed scheduling can be modeled as a multi-armed bandits model, the current study only designs a seed scheduling algorithm for coverage-guided fuzzing. Therefore, this study designs a seed scheduling algorithm for directed fuzzing to solve the exploration and exploitation problem.

## Methods

To overcome the multi-targets and indirect call problems in distance calculation, we developed a dynamic seed distance calculation method. The methods identifies indirect calls in the program and dynamically calculates the distance when the seed reaches indirect call. Besides, we donate the basic block distance as the minimal of the distance from the current basic block to all the target basic blocks, and use minimal distance of executed basic blocks in trace as seed distance to overcome the target bias problem.

With this seed distance calculation method, we designed a seed scheduling algorithm based on the UCB algorithm. The seed scheduling algorithm can select the seed from a seed pool based on the seed distance and its rareness.

### Seed distance calculation

In this section, we proposed a new seed distance calculation to solve the indirect call problem and the target bias problem.

#### Indirect call

We use the following strategy to handle the indirect call problem: First, in the static analysis stage, we use the points-to analysis to identify the indirect call and its callee, and generate the corresponding call graph and inter-procedural control flow graph. Second, in the dynamic analysis stage, we prune the infeasible path introduced by points-to analysis through seed distance calculation. Given a seed whose shortest path contains a specific indirect call site and the target callee, denoted as an **indirect call edge**, the more testcases generated by the seed reach the indirect call site but do not call the target callee, the more likely that the path containing the indirect call edge is an infeasible path, and we should increase the seed distance so that the fuzzer pays less attention to this seed. In other words, as the number of seed selections increases, the seed distance increases if the testcases generated by the seed cannot hit the indirect call edge.

#### Function distance

The function distance is calculated on the call graph. When the shortest path contains an indirect call edge, in static distance calculation stage, we identify the indirect call edge and store the current function distance as the distance from current function to the indirect caller. In 3.1.1, we consider that the seed distance will increase as the number of times the seed is selected increases; therefore, in the dynamic distance calculation stage, we multiply the number of times with the current function distance calculated in static distance calculation stage and add it with the distance from indirect caller function to target function as the function distance.

Formally, at time *t*, given a function *n*, the function distance *d*_*F*_(*t*, *n*, *T*_*f*_) from function *n* to the target function set *T*_*f*_ is defined as follows.

$$\begin{array}{c} \begin{array}{c} d_{F}\left(t,n,T_{f}\right)=\{\begin{array}{cc} \text{inf}., & \forall t_{f}\in T_{f},R\left(n,T_{f}\right)=\emptyset \\ {\text{min}}_{t_{f}\in T_{f}}D\left(n,t_{f}\right)), & IC(R\left(n,t_{f}\right))=\emptyset \;\\ {\text{min}}_{t_{f}\in T_{f}}(\sqrt{a}\times D\left(n,f_{icall}\right)+D\left(f_{icall},t_{f}\right)), & IC(R\left(n,t_{f}\right))\neq \emptyset \end{array} \end{array} \end{array}$$ (1)

where “inf.” means there is no reachable path from function n to all functions in the target function set *T*_*f*_, in practice, “inf.” is 65535. *R*(*n*, *t*_*f*_) is the reachable path set from function n to function *t*_*f*_ in the call graph. *IC*(*P*) is the set of untouched indirect call edges in nonempty path set P at round t. *f*_*icall*_ means the callee of the nearest untouched indirect call edge to current function. *D*(*n*, *t*_*f*_) is the Dijkstra distance from function *n* to function *t*_*f*_ in the call graph. *a* is the number of times that seed is selected and the testcases generated from the seed can reach the indirect call site at the end of round *t*.

We can see from Eq 1 that if a increases, the probability that the seed can generate the testcases whose trace contains indirect call edge decreases, the seed distance increases and the fuzzer will less frequently select the seed in the future. In addition, to eliminate the preference for the target function, we use a minimum function to calculate the distance from the current function to all the target functions.

#### Edge distance

The edge distance is calculated on the inter-procedural control flow graph. This indicates the shortest distance from the current edge to the basic target block. Similar to the function distance, at time *t*, given an edge *e* in the inter-procedural control flow graph and its destination basic block *n*, the distance *d*_*E*_(*t*, *e*, *T*_*b*_) to target basic block *T*_*b*_ is defined as follows.

$$\begin{array}{c} \begin{array}{c} \begin{array}{cc} & d_{E}\left(t,e,T_{b}\right)=\\ & \left\{ \begin{array}{cc} \text{inf}., & \forall t_{f}\in T_{f},R(n_{o},T_{b})=\emptyset \\ {\text{min}}_{n_{o}\in N_{o},f_{o}\in F_{c}\left(n_{o}\right)}\left(D(n,n_{o})+c\times d_{F}(t,f_{o},T_{f})\right), & IC(R(n_{o},T_{b}))=\emptyset \\ {\text{min}}_{n_{o}\in N_{o},f_{o}\in F_{c}\left(n_{o}\right)}(\sqrt{a}\times D(n,n_{o})+c\times d_{F}(t,f_{o},T_{f})), & IC(R(n_{o},T_{b}))\neq \emptyset \; \end{array}\right. \end{array} \end{array} \end{array}$$ (2)

where *N*_*o*_ is the set of exit basic block of the current function and *F*_*c*_(*n*_*o*_) is the function called in basic block *n*_*o*_. “inf.” indicates that there is no reachable path from basic block n to all exit blocks in *N*_*o*_, or there is no reachable path from the current function to all functions in the target function set *T*_*f*_, and its value is the same as “inf.” defined in Eq 1. *R*(*a*, *b*) is the reachable path set from *a* to *b* in the inter-procedural control flow graph.*IC*(*P*) is the set of untouched indirect call edges in nonempty path set *P* at round *t*. *f*_*icall*_ means the callee of the nearest untouched indirect call edge to current function. *D*(*a*, *b*) is the Dijkstra distance from basic block *a* to basic block *b* in the inter-procedural control-flow graph, *d*_*F*_(*t*, *a*, *T*_*f*_) is the function distance from function *a* to target function set *T*_*f*_ at round *t*. *a* is same as defined in Eq 1.

#### Seed distance

The seed distance measures the priority of the seed for the fuzzer. After defining the function distance and edge distance, we define the seed distance of seed *s* at round *t*.

$$\begin{array}{c} \begin{array}{c} seeddistance(s,t)=\underset{e\in edgecov(P,s)}{\text{min}}d_{E}(t,e,T_{b}) \end{array} \end{array}$$ (3)

where *edgecov*(*P*, *s*) represent the edge coverage with seed *s* under program *P*.

### Seed scheduling algorithm

To manage the exploration and exploitation problem in seed scheduling, we designed a new reward function and introduced the UCB algorithm for seed scheduling.

Specifically, we defined the score of the edge. Intuitively, the smaller the edge distance, the higher the score. At the same time, we also need to consider edges having few hit counts (denoted as the **rareness** of edge), because these edges represent program states that are less explored, and we should pay more attention to these program states. Therefore, at round *t*, given the target basic block set *T*_*b*_, the score *S*(*e*, *t*) of edge *e* is defined as

$$\begin{array}{c} \begin{array}{c} S(e,t)=\frac{1}{d_{E}(t,e,T_{b})\times \text{log}\left(hit(e,t)\right)} \end{array} \end{array}$$ (4)

where *d*_*E*_(*t*, *e*, *T*_*b*_) is edge *e*’s distance at round *t*. *hit*(*e*, *t*) means the hit count of edge *e* at round *t*. Since the hit count of edge *e* is usually extremely high in fuzzing, we used log(*hit*(*e*, *t*)) to represent the rareness of edge *e*.

With the definition of the edge score, we can define the reward of the seed *s* at round *t*. We expect that if the testcases mutated from the seed hit an edge having a high score, then the seed’s estimated reward should be equally high. However, the mutation count of a seed is determined by seed distance, which influences the reward for seed selection. In the classic greybox fuzzer AFLGo, the mutation count of a seed is calculated as follows:

$$\begin{array}{c} \begin{array}{c} energy(s)={energy}_{AFL}(s)\times a^{seeddistance(s,t)-0.5}\times e^{T} \end{array} \end{array}$$ (5)

where *energy*(*s*) determines the mutation count of seed *s*. *T* is a distance-independent parameter of the simulated annealing algorithm, *energy*_*AFL*_(*s*) is a distance-independent parameter that determines the mutation count of seed *s* in AFL-like fuzzers. To eliminate the seed distance bias, we define the reward for choosing seed s at round t as follows:

$$\begin{array}{c} \begin{array}{c} Reward(s,t)=\underset{e\in edgecov(P,\;I),\forall I\in I_{s,t}}{\text{max}}\frac{S(e,t)}{c\times a^{seeddistance(s,t)}} \end{array} \end{array}$$ (6)

where *I*_*s*,*t*_ is the testcase set mutated by seed s at round t. *edgecov*(*P*, *I*) is the edge coverage of program *P* with input *I*. The constant *c* is the seed distance bias, which is *a*^−0.5^ according to Eq 5. *a* is 2 according to AFLGo.

We introduced the UCB algorithm to address the exploration and exploitation problem with the seed reward defined above. The standard form of the UCB algorithm is.

$$\begin{array}{c} \begin{array}{c} R(a_{t})=Q(a_{t})+U(a_{t}) \end{array} \end{array}$$ (7)

In the original UCB algorithm, reward is a constant variable. However, in seed scheduling, the reward becomes smaller during fuzzing because the hit count increases [21]. Therefore, we used the weighted average value to calculate the estimated average reward:

$$\begin{array}{c} \begin{array}{c} Q(s,t)=\frac{Reward(s,t)+w\times Q^{\prime }(s,t)\times \sum_{i=0}^{t^{\prime }}w^{i}}{1+w\times \sum_{i=0}^{t^{\prime }}w^{i}} \end{array} \end{array}$$ (8)

*t*′ is the number of times seed *s* has been selected at the end of round *t*. *Q*(*s*, *t*) donates the estimated average reward for choosing seed *s* at round *t*. *Q*′(*s*, *t*) is the estimated average reward for choosing seed *s* at the last round. The upper confidence bound *U*(*s*, *t*) is

$$\begin{array}{c} \begin{array}{c} U(s,t)=\sqrt{\frac{2log(t)}{t^{\prime }}} \end{array} \end{array}$$ (9)

In addition, if this seed quantity is good but the testcases mutated by this seed are not good, the reward for choosing this seed is not high enough. For example, if a seed passes a complex constraint check and is close to the target, it must be considered important. However, the testcases mutated by this seed cannot pass this check because the constraint check is complex, and according to Eq 6, the reward for selecting the seed is not high. Therefore, we modified the score of seed selection, adding seed information, which names seed importance to calculate the score of seed scheduling, and the final score of selecting seed s at the end of round t, denoted as *Score*(*s*, *t*), is calculated as

$$\begin{array}{c} \begin{array}{c} SeedImportance(s,t)=\sqrt{\frac{\sum_{e\in edgecov(P,s)}{\left(\frac{1}{log\left(hit(e,t)\right)*d_{E}(t,e,T_{b})}\right)}^{2}}{\left|edgecov(P,s)\right|}} \end{array} \end{array}$$ (10)

$$\begin{array}{c} \begin{array}{c} Score(s,t)=SeedImportance(s,t)\times (Q(s,t)+U(s,t)) \end{array} \end{array}$$ (11)

## Implemention

**Fig 4:**
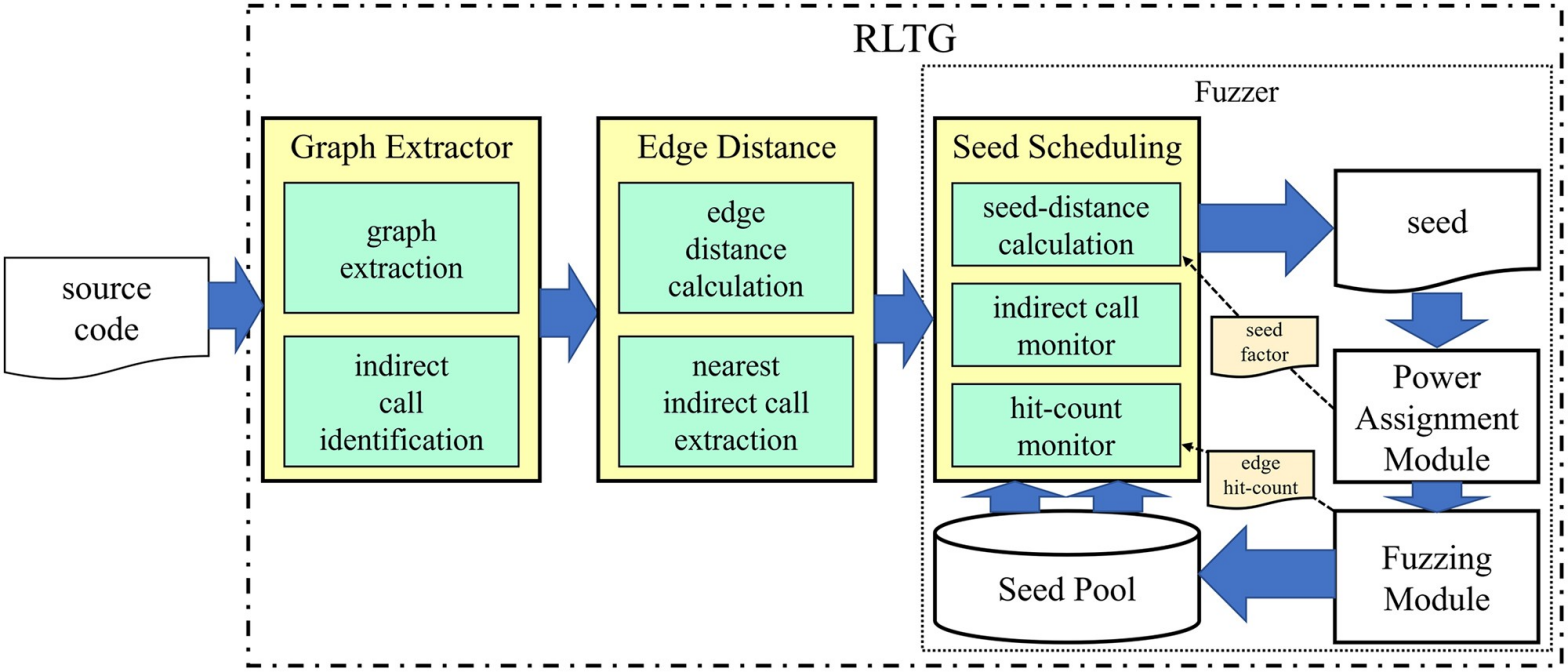
The architecture of RLTG.

To verify our enhancement in directed greybox fuzzing, we designed our prototype RLTG. As shown in Fig 4, RLTG consists of three modules: graph extractor, edge distance calculation, and seed scheduling. The graph extractor constructs a call graph and an inter-procedural control flow graph. Both the call graph and the control flow graph are used to calculate the edge distance. With the help of the edge hit count information and edge distance, seed scheduling uses a dynamic distance calculation algorithm and a UCB-based seed scheduling algorithm to select the next seed.

### Graph extractor

The graph extractor constructs a call graph and an inter-procedural control flow graph. While constructing the call graph, we used the points-to analysis to identify the indirect call and its callee in the program. The points-to analysis [22] determines whether two pointers are aliases of each other by calculating whether the intersection of the two pointers’ points-to sets is null. The points-to set refers to the set of elements that contains all the variables the pointer points to.

This study adopted the Andersen algorithm [23], which is a flow-insensitive and context-insensitive points-to analysis method, for its efficiency and accuracy in identifying all the function pointers and their callees After the points-to analysis, we saved the (call site, callee) tuple and constructed the call graph and inter-procedural control flow graph based on Low Level Virtual Machine(LLVM).

### Edge distance calculation

The edge distance calculation module calculates the distance of all the reachable edges in the static analysis. The edge distance calculation calculates the ID of each edge and the corresponding edge distance using the basic block information and shortest path information. It is divided into two parts: a) for a path that does not contain indirect calls along the shortest path, the distance of each edge in the path is obtained from the Dijkstra distance from the exit basic block of the edge to the target basic block. b) The shortest path contains an indirect call. First, we use a call graph and an inter-procedural control flow graph to find the shortest path between the exit basic block of the edge and the target basic block. We then check whether the successor edge of each edge along the shortest path is an indirect call edge. If so, the distance of the current edge is calculated as the distance from the exit basic block of edge to the indirect call site basic block. Otherwise, the edge distance is calculated according to a).

### Seed scheduling

The seed scheduling algorithm monitors executed indirect calls in the program, dynamically calculates the seed distance from the edge distance calculated in static analysis and calculates the score of each seed in the seed pool based on the hit count of the edge and the seed scheduling status. The seed distance calculation algorithm is shown in Algorithm 1. In lines 4–8, RLTG uses the indirect call information and edge distance obtained from the static analysis to dynamically calculate the distance of the edge executed by the current seed, thereby calculating the seed distance.

**Algorithm 1** SeedDistance(): Seed Distance Calculation

**Input:** s, the seed to be calculated its distance; indirect_call, the array contains edge index and its neatest indirect call edge index; distance, the array contains distance information; a, the number of times the seed s is selected for fuzzing

**Output:** seed_distance, the seed distance for current seed

1: seed_distance = 0

2: **while** i < MAP_SIZE **and** seed not hit edge i **do**

3:  edge_distance = 0; j = i;

4:  **while** !indirect_call[j] **do**

5:   edge_distance += a * distance[j]

6:   j = indirect_call[j]

7:  **end while**

8:  edge_distance += distance[j]

9:  **if** seed_distance > edge_distance **then**

10:   seed_distance = edge_distance

11:   i++

12:  **end if**

13: **end while**

In the seed scheduling algorithm, as shown in Algorithm 2, we first calculate the estimated average reward of the current seed according to the edge mutated by the current seed and its distance. Second, we calculate the seed score using the seed scheduling times and the number of times the seed was chosen. Moreover, we calculate the seed importance according to the edge executed by the seed, select the seed with the highest seed score as the seed selected in the current round for fuzzing and update the seed information, including the number of times the seed was selected, and the average estimated reward of the seed. After a round of fuzzing, RLTG records the edge hit count of the testcases generated by the mutation of the current seed and updates the indirect call information in the program.

**Algorithm 2** SeedSchedule(): Seed Scheduling Algorithm

**Input:** Q, seed queue; h, hit count information; n, seed scheduling times

**Outout:** s, seed to be fuzzed

1: q = Q; max = 0;

2: **while** q.next ≠ null **do**

3:  seeddistance = SeedDistance(s)

4:  seedreward = Reward(s, h, seeddistance)

5:  score = Score(s, seedreward, h, n)

6:  **if** score > max **then**

7:   s = q

8:  **end if**

9:  q = q.next

10: **end while**

## Evaluation

To verify our idea, we designed a prototype RLTG based on AFLGo and evaluated it on several benchmarks to answer the following research questions.

**RQ1** Can RLTG trigger vulnerabilities faster than the state-of-the-art fuzzers in the multi-targets problem?

**RQ2** Can the seed scheduling algorithm perform handle the exploration and exploitation problems in directed greybox fuzzing?

**RQ3** Can the dynamic seed distance calculation scheme handle the target bias problem in the multi-targets scenarios?

**RQ4** Can the dynamic seed distance calculation scheme fix the indirect call problem in distance calculation?

**RQ5** What is the overhead of the RLTG?

### Evaluation setup

In the experiment, we used Magma to evaluate the performance of multi-targets directed fuzzing. Magma is a mainstream fuzzing benchmark that contains multiple CVE vulnerabilities in various real-world software such as libpng, libtiff, libsndfile and SQLite. Magma identifies and marks the location of patches corresponding to CVE vulnerabilities in the latest software repo; thus, we can identify the target by targeting every basic block in the project that contains a magma mark. The effectiveness of the edge-distance-based seed distance calculation method proposed in this study was verified by evaluating the directed fuzzing performance of RLTG for multi-targets detection. Besides, we used binutils, jasper, libming, lrzip, lame, zziplib, those real-world programs evaluated in previous work [4, 24], to evaluate the performance of Seed Scheduling Strategy in RLTG.

**Baseline.** RLTG was implemented based on the latest version of AFLGo, whereas the indirect call recognition was implemented based on the open-source points-to analysis framework SVF [25]. RLTG* is a clone of RLTG without seed scheduling algorithm proposed in this study. This study compared RLTG with the directed greybox fuzzer AFLGo to show the performance of the dynamic seed distance calculation scheme and seed scheduling algorithm. The technical details of AFLGo are described in Chapter 2. Since AFLGo is developed on AFL, We also compared RLTG with coverage-guided greybox fuzzer AFL [26] and the state-of-the-art AFL-based coverage-guided greybox fuzzer FairFuzz [27]. We also planed to compare with other directed fuzzing tools. However, Hawkeye is not open source. The weakest precondition used in Beacon [24] doesn’t support multi-targets yet. Savior [28] utilizes UBSan to identify the targets and uses symbolic execution to reach the targets. Since its not directed greybox fuzzer and its applicable scenarios are different, we support it is orthogonal to our approach. In the experiment, we used time-to-exposure(TTE) to show the time when the fuzzer first finds the testcase that can trigger the vulnerability.

**Evaluation setup.** All experiments were conducted on a computer with Intel^®^ Xeon^®^ Gold 6154, containing 72 logical cores and 512 GB of memory. The operating system was Ubuntu 18.04. Considering that the initial seed pool can influence fuzzing performance, we used the null seed or testcases provided by AFL to test the fuzzer. In the experiments, all programs were repeated 10 times within 12 hours.

### Crash reproduction capability against multi-targets

We compared the crash reproduction capability of RLTG with AFLGo against magma to show the efficiency of RLTG against multi-targets directed fuzzing. Because magma records the number of testcases that satisfy the vulnerability trigger constraint through magma_log, in our experiments, we treat the basic block that calls magma_log as the target basic block. The experimental results are listed in Table 1.

**Table 1:** Comparison of RLTG, AFLGo, and AFL with 10 repeated experiments of vulnerability reproduction against magma.

Target		*μ*TTE				
Program	CVE num.	AFL	FairFuzz	AFLGo	RLTG*	RLTG
libpng	CVE-2015-8472	<1m	<1m	<1m	<1m	<1m
	CVE-2013-6954	T.O.	2h19m	T.O.	8h37m	1h24m
libsndfile	CVE-2017-6892	14m	28m	18m	3h50m	<1m
	Commit a8ab5b3	52m	T.O.	1h4m	58m	59m
	CVE-2017-8363	8h18m	T.O.	T.O.	T.O.	59m
tiffcp	CVE-2016-10270	11h57m	5h22m	11h6m	6h18m	3h36m
	CVE-2016-3658	T.O.	8h52m	T.O.	T.O.	1h47m
	CVE-2017-11613	T.O.	T.O.	T.O.	T.O.	5h54m
tiff_read_rgba_fuzzer	CVE-2015-8784	T.O.	T.O.	T.O.	T.O.	10h6m
	CVE-2016-10270	25m	4m	36m	2h53m	2h23m
	CVE-2016-3658	8h50m	24m	3h35m	3h8m	1h35m
	CVE-2017-11613	4h51m	6h53m	11h2m	9h43m	6h26m
sqlite_fuzzer	CVE-2015-3414	8h36m	6h32m	9h48m	6h34m	6h24m
	CVE-2019-19926	10h33m	8h8m	9h34m	6h43m	6h33m
	CVE-2019-20218	11h13m	6h17m	10h30m	10h31m	10h27m

T.O. means that within 12 hours, the fuzzer could not reproduce the crash.

In 12 hours, AFLGo could only reproduce 9 crashes respectively while RLTG can reproduce 15 crashes, 8 of them in 4 hours. RLTG can reproduce vulnerabilities faster than AFLGo in 80% of the CVE-identified vulnerabilities and achieve 6.9x speedup on average than AFLGo. Besides, compared with the state-of-the-art fuzzer FairFuzz, RLTG can reproduce vulnerabilities faster than FairFuzz in 73.3% of the CVE-identified vulnerabilities. The results show that our prototype RLTG is efficient in multi-targets directed fuzzing. Compared with AFLGo, RLTG could treat the target evenly and could handle the exploration and exploitation problem with distance and code coverage information.

Besides, compared with the base platform AFLGo, RLTG*, a prototype with dynamic seed distance calculation proposed in this paper, reproduced one more bug than AFLGo, and achieved 1.05x speedup on average than AFLGo. In 70% of the cases, RLTG* reproduced the bugs with less time. The results show that our proposed dynamic distance calculation method improves the performance towards multi-targets scenarios. But even with the proposed seed distance calculation methods, RLTG* still suffered from the exploration and exploitation problem in fuzzing process. During fuzzing process, unlike RLTG, RLTG* would like to choose one vulnerability as target and neglect other targets.

However, Table 1 shows that in some CVE-identified vulnerabilities, time cost of crash reproduction is higher than AFL and AFLGo. In CVE-2016-10270, AFLGo could trigger the bug with less time than RLTG, we thought that the distance towards other bugs affect the efficiency. On magma, the seed pool contains several seeds close to the different targets, RLTG and RLTG* treat the different targets equally, the ability towards multiple bugs really affects the vulnerability detection towards specific bug, so sometimes the time cost to specific CVE-identified vulnerabilities of RLTG and RLTG* were a bit higher than AFL and AFLGo.

### Balanced targets detection capability against multi-targets

**Fig 5:**
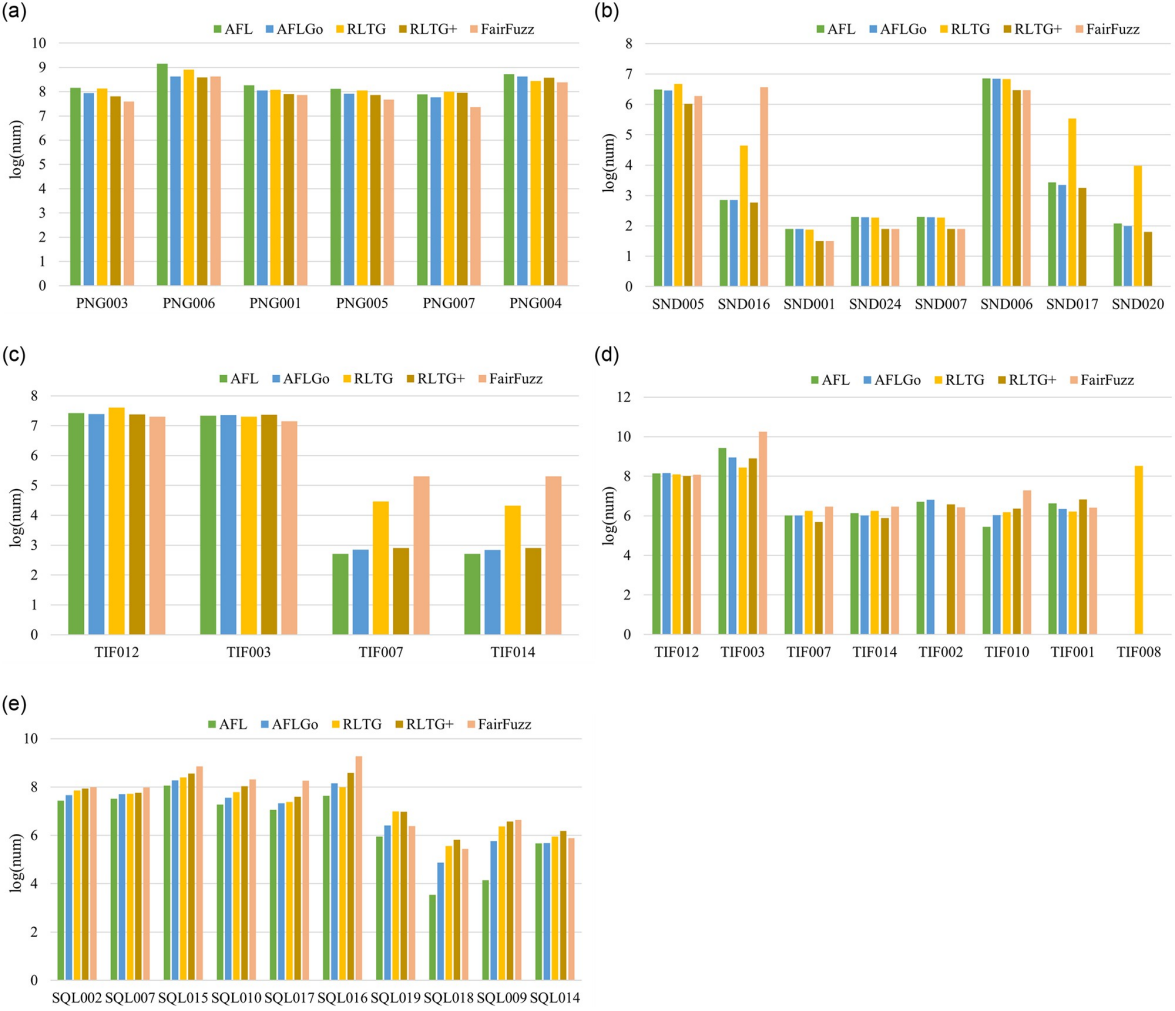
Number of testcases reaching the targets in RLTG, AFLGo, and AFL against magma. All fuzzing times were cut off at 12 hours. The X-axes indicates the vulnerability ID in magma. The Y-axes indicates the hit count in the vulnerability basic block A.libpng B.libsndfile C.tiffcp D.tiff_read_rgba_fuzzer E.sqlite_fuzzer.

To verify that the well-balanced multi-targets fuzzing capability of RLTG and RLTG*, we recorded the number of testcases that reached the target basic block. The results are shown in Fig 5.

As shown in Fig 5, compared with AFLGo and AFL, RLTG reaches more target basic blocks. In tiff_read_rgba_fuzz and sqlite_fuzzer, RLTG reached more targets. From the figure, we can see that the number of testcases that reached the target basic block seemed more balanced for RLTG than for AFL and AFLGo.

Besides, we calculate the deviation of the number of testcases that reach different target. Since for fuzzer to mutate testcases that reach the target is not equally easy for different targets, simply calculating the standard deviation of the number of testcases reaching the targets may enhance the impact of those easy-to-reach targets. Instead, we quantified the fuzzer’s multi-targets fuzzing ability by calculating the standard deviation of the logarithm of the number of testcases that reached the target basic block. The results are presented in Table 2.

**Table 2:** Standard deviation of the number of testcases reaching different vulnerability sites in different programs.

Program	Standard Deviation				
	AFL	FairFuzz	AFLGo	RLTG*	RLTG
libpng	1.08	1.21	0.72	0.67	0.62
libsndfile	28.06	32.81	28.12	27.00	27.94
tiffcp	21.82	3.71	20.47	19.94	9.42
tiff_read_rgba_fuzzer	11.48	11.24	8.39	8.03	5.59
sqlite_fuzzer	20.60	15.23	11.84	8.13	7.62

The Table 2 shows that the seed distance calculation scheme can help enhance the multi-targets fuzzing capability. Compared with AFLGo, RLTG* and RLTG can treat all the targets equivalently than AFLGo. Without proposed seed distance calculation scheme, AFLGo suffered from the target bias problem, it preferred the targets with shorter reachable path thus the deviation is much higher than RLTG and RLTG*. However, RLTG* still suffered from the exploration and exploitation problem in seed scheduling component, thus the the deviation of RLTG* is higher than RLTG.

### Seed scheduling performance

To verify our UCB-based seed scheduling algorithm is efficient in single-target directed fuzzing, we compared the crash reproduction capability of RLTG with RLTG*. The results are presented in Table 3.

**Table 3:** Compares to RLTG* against the real-world programs using 10 repeated experiments for vulnerability reproduction.

Target		*μ*TTE	
Program	CVE num.	RLTG*	RLTG
libming-0.4.7	CVE-2016-9827	57m	33m
	CVE-2016-9829	T.O.	7h45m
	CVE-2016-9831	1h2m	58m
	CVE-2017-7578	59m	1h13m
	CVE-2018-11728	5h26m	4h9m
	CVE-2018-11729	1h29m	1h58m
libming-0.4.8	CVE-2018-8807	T.O.	5h32m
	CVE-2018-8962	T.O.	3h18m
jasper	CVE-2015-9560	T.O.	8h32m
cxxfilt	CVE-2016-4487	1h22m	1h7m
	CVE-2016-6131	T.O.	8h26m
lrzip	CVE-2017-8846	T.O.	6h1m
	CVE-2018-11496	<1m	<1m
lame	CVE-2017-15045	2m	1m
	CVE-2017-15046	8h47m	3h12m
zziplib	CVE-2018-6381	1h36m	49m

T.O. means that within 12 hours, the fuzzer could not reproduce the crash.

We observed that RLTG* could not reproduce 6 out of the 16 vulnerabilities within 12 hours, whereas RLTG could reproduce all of them, and it could reproduce 10 of them within 4 hours. RLTG could reproduce vulnerabilities faster than RLTG* in 75% of the CVE-identified vulnerabilities and achieve a 1.65x increase in speed on average compared with RLTG*. In the worst case, RLTG found bugs with 20min delay, while in 87.5% of the cases, RLTG reproduced crashes faster than RLTG*. In seed scheduling phase, RLTG wasted some time in choosing same-distance seeds and wasted some energy in testing them, so sometimes RLTG’s performance would slightly worse than RLTG*. The results showed that the seed scheduling algorithm proposed in this study could help address the exploration and exploitation problem in directed greybox fuzzing and enhance the performance.

### Overhead

To quantify the system overhead of the seed scheduling algorithm in RLTG, we first analyze the possible system bottlenecks in RLTG. We believe that the seed selection algorithm and the dynamic seed distance calculation method are the main bottlenecks in RLTG. Therefore, we tested the execution speed of RLTG, and compared it with RLTG+, which does not use the seed selection algorithm and the dynamic seed distance calculation scheme proposed in this study, to visualize the overhead of the seed selection algorithm and the dynamic seed distance method. Besides, we also compared the testcase execution per second of RLTG with RLTG* to show the overhead of our proposed seed distance calculation schema.

**Fig 6:**
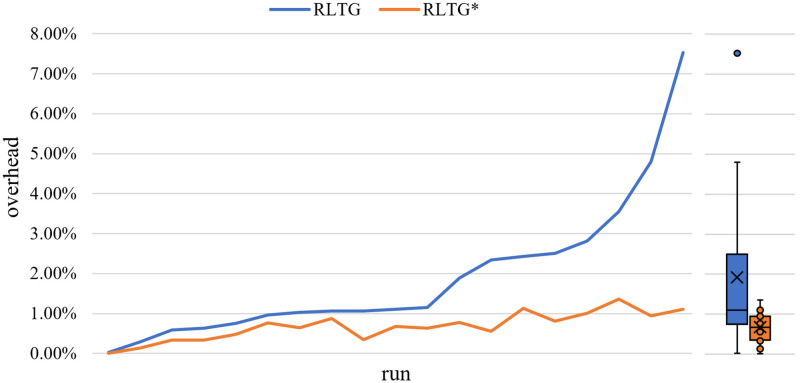
Overhead of RLTG compared to RLTG+ on benchmark. The x-axis represents each program in the benchmark. The y-axis represents the difference in the number of testcases executed per second between RLTG and RLTG+ as a percentage of the number of testcases executed per second in RLTG+.

The final result is shown in Fig 6, according to the experimental results, the performance overhead of the seed scheduling algorithm and the dynamic seed distance calculation scheme based on edge distance is small. The average performance gap between RLTG and RLTG+ was approximately 2%, with a maximum value of 7.7%. The overhead of seed distance proposed in this paper is slight, with approximately 0.7% and maximum overhead of this execution is 1.4% It is because the main bottlenecks of fuzzer overhead is target execution, and we update the seed distance only during seed scheduling, thus the overhead of seed distance calculation is small. We found that in fuzzing process, seed needs thousands of times to be mutated in a fuzz round, so seed scheduling take little time in fuzzing process and the overhead of seed scheduling algorithm is small. Therefore, we conclude that the dynamic seed distance calculation scheme and seed scheduling algorithm achieved good results with a low performance overhead.

### Case study

We used CVE-2016-9560 to illustrate the efficiency of the dynamic distance calculation methods towards indirect call problem. In the experiment, we chose two unique seeds: one was a jpc file with corrupted metadata, denoted as **corrupted-jpc-seed**, and the other was a standard bmp file, denoted as **bmp-seed**. We kept the size of the seed pool to 2 and observed the change in seed distance and seed score over time.

**Fig 7:**
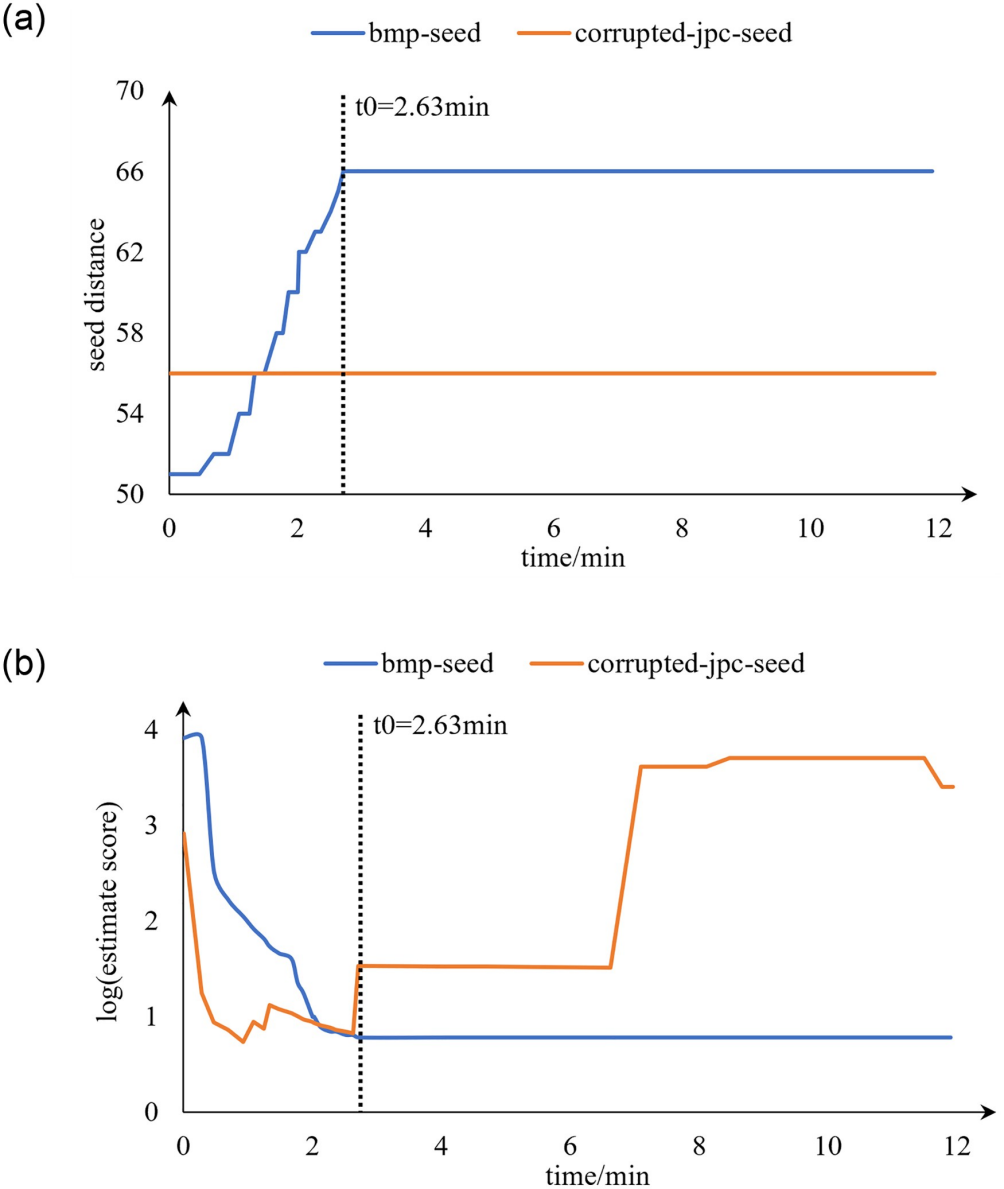
Seed distance and estimated score of two unique seeds in RLTG.

The experimental results show that the edge-distance-based dynamic distance calculation method can address indirect calls in the program. At the initial stage, As shown in Fig 7(a), because the bmp-seed calls bmp_decode() by the function pointer and bmp_decode() cannot reach the target, the seed distance is 51, which is the distance from the indirect call site to the target. However, the distance of the corrupted-jpc-seed is higher than that of bmp-seed because the corrupted-jpc-seed cannot pass the input format check. As a result, in the initial stage, RLTG chooses the bmp seed as the fuzz. However, the testcases mutated by bmp-seed do not call jpc_decode(), and its seed distance increases. As shown in Fig 7(b), after 152 s, the fuzzer chooses the corrupted-jpc-seed and generates testcases called jpc_decode(), and the seed estimated reward becomes larger than that of bmp-seed; thus, from then on, the fuzzer usually chooses the corrupted-jpc-seed and finds testcases that are closer to the target.

In addition, we chose several operators to evaluate the influence of seed quality on the distance calculation. As mentioned in Chapter 3, seed quality is quantified by the number of times that the seed is selected and the number of testcases generated from the seed that go to the indirect call site at the end of round t, so we used three typical operators to observe the influence. The results are presented in Table 4. We can see from the table that the most efficient operator is the square, so we set the factor to $\sqrt{a}$.

**Table 4:** Different factors in the performance of RLTG.

Operator	*a*	log(*a*)	$\sqrt{a}$
*μ* **TTE**	T.O.	T.O.	8h32m

T.O. means that within 12 hours, the fuzzer could not reproduce the crash.

### Threats to validity

The internal validity of this study depends on several factors: 1. The validity of the static analysis results. RLTG used the static analysis tools SVF and LLVM to calculate the seed distance. The accuracy of these tools can also affect the results. To improve scalability, other static analysis tools can also be used to calculate the seed distance; 2. The effectiveness of hyperparameter selection. Different hyperparameters may affect the performance of the final seed-scheduling algorithm; 3. The hash collision of the edges in the edge-distance calculation has a significant impact on the result. In the follow-up to this study, a new hash calculation method will be introduced to alleviate the edge hash collision problem.

Although the method proposed in this paper was verified on several library programs and real-world programs, the experimental results still need to be verified in terms of external validity on additional projects.

## Related work

### Coverage-guided greybox fuzzing

In recent years, researchers have continued to innovate, and have successively launched mainstream fuzzing tools such as AFL [26], LibFuzzer [29], and AFL++ [30] to detect vulnerabilities in software. Based on these fuzzers, researchers proposed several optimization methods, finding many vulnerabilities in display software.

Some researchers have introduced symbolic execution techniques into coverage-guided fuzzing to help fuzzer bypass complex path constraints. Such techniques are often referred as hybrid symbolic execution. Driller [31] combines symbolic execution with fuzzing. When the fuzzer encounters a check, the symbolic execution tool is used to collect constraints and solve the constraints, assisting fuzzer in passing the check; QSYM [32] uses dynamic binary translation technology to reduce the overhead of the symbolic execution engine translating binary codes into intermediate expressions. In addition, for over-constrained problems, QSYM uses the most recent constraint in the path constraints to solve the problem, which improves the efficiency of symbolic execution.

In addition, some researchers optimize fuzzer performance in other aspects. Observing that the throughput of mutation operators varies, MOPT [33] uses particle swarm optimization to optimize the probability of using mutation operators, which can effectively improve the mutation efficiency of greybox fuzzing. Some researchers use dynamic data flow analysis methods such as dynamic taint analysis to identify key bytes in the input and mutate the critical bytes to improve fuzzing performance. Angora [34] uses dynamic taint analysis to perform byte-level taint tracking on the input and mutate critical bytes; GreyOne [35] uses a lightweight taint analysis method to identify critical bytes for efficient fuzzing. Introducing the above-mentioned greybox fuzzing technology applied to coverage guidance in directional greybox fuzzing can reduce the complexity of directional fuzzing testing and improve fuzzing performance.

### Directed greybox fuzzing

Several researchers optimize the directed greybox fuzzing by pruning input in directed greybox fuzzing. FuzzGuard [36] trains a multi-layer convolutional neural network model, predicts whether a given input can reach the target through the neural network, and filters those unreachable inputs. WindRanger [37] uses reachability analysis and dynamic clipping to clip unreachable testcases during fuzzing; Beacon [24] uses program slicing and the weakest precondition to prune input. Such techniques can be integrated with the method proposed in this paper to improve the efficiency of directed fuzzing.

On the other hand, some researchers extract more information based on the characteristics of targets and design corresponding directed strategies to achieve efficient directed greybox fuzzing. SeededFuzz [38] utilizes various program analysis techniques to select and generate the seeds of the initial seed pool to implement directed fuzzing. However, our method selects seeds from the existing seed pool in the fuzzing process, so there is a significant difference between our work and SeededFuzz. ParmeSan [39] uses the sanitizer information to interpolate specific basic blocks that may trigger bugs and design a fuzzer for sanitizer. UAFuzz [11] designs a sequence-guided directed fuzzer because UAF vulnerability is triggered in s specific order. CAFL [12] uses the sequence information of the target and data constraints to improve the orientation efficiency of fuzzing.

### Seed scheduling

Many people have studied and modified the seed scheduling method in fuzzer and made much progress. CollAFL [40] proposes a new hash algorithm to alleviate the hash collision problem in large-scale software fuzzing and designs a seed selection method for edge adjacency. AFLFast [41] models the fuzzing process as a Markov chain, focusing the fuzzer’s attention on seeds that execute more low-frequency paths. By evaluating the coverage characteristics of testcases mutated by seeds with information gain, Entropic [42] argues that seeds with higher information gain should be easier to fuzzed. Ecofuzz [43] treats coverage-guided greybox fuzzing as an exploration and exploitation problem, and its reward is defined as the transition probability of the seed finding a new path. Based on this idea, Ecofuzz designs a seed scheduling and energy allocation algorithm for coverage-guided greybox fuzzing based on the multi-armed bandits model. Wang [21] aims at the fuzzer design problem under the multi-coverage standard. The reward of seed scheduling is determined by the number of the rarest edges hit in the testcase generated by the seed. He designs the seed scheduling with a multi-armed bandits model based on the reward. The algorithm can better solve the problem of seed explosion in the gray-box fuzzing of multi-granularity coverage standards. However, the objective function of directional fuzzing is different from that of coverage-guided fuzzing tools, so the existing methods cannot solve the contradiction of different directional targets when faced with different directional targets.

## Conclusion

In this paper, we proposed a new seed scheduling method and a new seed distance calculation method. Based on this idea, we designed a new directed greybox fuzzer RLTG. The design idea of RLTG is to balance the detection of each target in multi-targets directed greybox fuzzing and solve the exploration and exploitation problems in directed fuzzing. RLTG can achieve well-balanced multi-targets directed greybox fuzzing and faster vulnerability detection through the dynamic distance calculation method and the seed scheduling algorithm. The experimental results show that compared with the mainstream director fuzzer, RLTG can reach the target faster, and in the multi-targets directed fuzzing experiment, the fuzzing between each target is more balanced. It can be applied to several research fields such as 1-day vulnerability detection.
